# Exploring cadmium stress responses in sisal roots: Insights from biochemical and transcriptome analysis

**DOI:** 10.1371/journal.pone.0288476

**Published:** 2023-11-29

**Authors:** Jing Li, Lifang Ge, Ping Liu, Zhaoxue Huang, Shibei Tan, Weihuai Wu, Tao Chen, Jingen Xi, Xing Huang, Kexian Yi, Helong Chen

**Affiliations:** 1 National Key Laboratory of Green Pesticide, Key Laboratory of Green Pesticide and Agricultural Bioengineering, Ministry of Education, Center for R&D of Fine Chemicals of Guizhou University, Guiyang, China; 2 Environment and Plant Protection Institute, Chinese Academy of Tropical Agricultural Sciences (CATAS), Haikou, Hainan, PR China; 3 Guangxi Subtropical Crops Research Institute, Nanning, PR China; 4 Sanya Research Institute, Chinese Academy of Tropical Agricultural Sciences, Haikou, China; Jeju National University, REPUBLIC OF KOREA

## Abstract

Sisal is a leaf fiber crop with a high integrated value and a wide range of uses in the application of soil remediation of heavy metal contamination. This study provides a preliminary understanding of how sisal responds to Cd stress and presents a theoretical basis for exploring the potential of sisal in the remediation of Cd-contaminated soils. In this work, the activities of the antioxidant enzymes (SOD, POD, and CAT) of sisal were measured by hydroponics with the addition of CdCl_2_·2.5H_2_O and different concentrations of Cd stress. Whole transcriptome sequencing (RNA-Seq) analysis was performed with lllumina sequencing technology, and qRT-PCR was conducted to verify the differential genes. The results obtained were as follows: (1) Short-term low concentration of Cd stress (20 mg/kg) had a transient promotion effect on the growth of sisal roots, but Cd showed a significant inhibitory effect on the growth of sisal roots over time. (2) Under different concentrations of Cd stress, the Cd content in sisal root was greater than that in sisal leaf, and Cd accumulated mainly in sisal roots. (3) With the increase of Cd stress concentration, the antioxidant enzyme catalase activity increased, peroxidase activity showed a decreasing trend, and superoxide dismutase showed a trend of increasing and then decreasing. (4) Transcriptome sequencing analysis detected 123 differentially expressed genes (DEGs), among which 85 genes were up-regulated and 38 genes were down-regulated. The DEGs were mainly concentrated in flavonoid biosynthesis and glutathione metabolism, and both processes had some regulatory effects on the Cd tolerance characteristics of sisal. This study elucidated the physiological, biochemical and transcriptomic responses of sisal under cadmium stress, and provided a theoretical basis for the ecological restoration function of sisal.

## 1. Introduction

Cadmium (Cd) is a widely distributed, highly polluting, and highly toxic heavy metal. Cd’s harmful effects on the agricultural environment mainly arise from its extremely high environmental mobility, persistence, and difficult degradation [1]. Primarily absorbed and accumulated by plant roots, Cd seriously affects the growth and development of plants and is transferred to other organisms through the food chain, consequently endangering the life and health of animals and humans [2]. High Cd concentration in soils negatively affects plant growth and agricultural productivity [3]. Cd is chiefly absorbed by and accumulated in plant roots, and this feature severely affects plant growth and development; Cd is subsequently transferred to other organisms through the food chain, thereby endangering the life and health of animals and humans [4–6]. Cd concentration at 2 mg/kg can decrease rice yields by 25%, and the same yield reduction is achieved at Cd levels of 4–13 mg/kg for spinach, soybean, and kudzu bar [7]. Zhou et al. (2016) showed that under Cd^2+^ stress, *Nicotiana tabacum* growth was inhibited, for which the symptoms included plant dwarfism, loss of green leaves, leaf detachment, and even death [8]. Moreover, long-term exposure to Cd^2+^ decreases tomato (*Solanum Lycopersicum*) yield by reducing the weight and number of fruits [9, 10].

Reducing heavy metal contamination in soils, ensuring the safety and yield of agricultural products, and promoting the sustainable and efficient use of land resources are issues that must be addressed urgently. In such a context, the prompt development of methods to decontaminate contaminated soil is vital. Phytoremediation is a promising and cost-effective technique for decontaminating soils tainted with heavy metals [11, 12]. Given its good ecological and economic benefits, phytoremediation is the predominant soil remediation technique at this stage [13]. Energy crops belong to a kind of phytoremediation and are characterized by high photosynthetic efficiency, large biomass, rapid growth, strong adaptability, and wide distribution. In the remediation of heavy metal-contaminated soil, such plants can provide biomass energy, so that the remediation work and energy production can be integrated [14]. This integration completes the transfer of heavy metals from the food chain to the energy plant chain, which not only ensures the safe production of food crops, but also solves the soil pollution problem; further, energy crops constitute a type of remediation plant with development potential [14].

Sisal, a leaf fiber plant of the genus Agave that grows in the tropics, is also a class of perennial, succulent, dry herbaceous plants. Sisal fiber is one of the most widely used and extensive hard fibers in the world today. Furthermore, sisal has a well-developed root system and is adaptable to soil, cold, drought-like high temperatures, wind resistance, and other characteristics. Given the large biomass and long growth cycle of sisal, its processed products are separated from the food chain, resulting in its advantageous application in the remediation of heavy metal-contaminated soil [15]. Shen et al. (2009) found that sisal H.11648 was more effective than the Mauritius variant for the accumulation of most heavy metals and had a stronger advantage for tailings pond remediation [16]. Wu et al. (2009) established that sisal had strong sorption of Pd and had some potential for remediation of Pd-contaminated soil [17]. Chen et al. (2007) established that the growth of sisal was not greatly affected in Cd 25 mg/kg contaminated soil, and could grow in Cd 50 mg/kg contaminated soil, but was severely poisoned in the Cd 100 mg/kg counterpart [18].

H.11648 was introduced from abroad in the 1960s, started to be promoted on a large scale throughout China in the 1970s, and has now become the main germplasm for sisal cultivation in the country [19]. With its high yield and high quality, H.11648 has played an important role in the development of the sisal industry in China [19]. In recent years, transcriptomics technologies have been rapidly developed to provide an accurate approach to gaining insight into the molecular mechanisms of plant and animal responses to abiotic stresses. Transcriptome sequencing (RNA-seq) has been widely used in a variety of plants such as rice [20], wheat [21], and oilseed rape [22] to better understand the response mechanisms of plants under Cd stress and reveal the Cd contents in different plants. Apprehending more response mechanisms of plants under Cd stress and revealing the molecular mechanisms of Cd translocation, accumulation, and detoxification in different plants are necessary to provide a theoretical basis for genetic engineering to modify Cd-tolerant plants. Therefore, in this study, the transcriptomic and biochemical analyses of root Cd stress were conducted using H. 11648 as the subject of study. The molecular response mechanism of sisal roots to this stress and the related antioxidant enzyme activities were investigated thereby providing a theoretical basis for the environmental remediation of Cd contamination in sisal soils.

## 2. Materials and methods

### 2.1 Plant material, growth, and handling conditions

Sisal seedlings (variety H.11648) were selected from the sisal plant nursery of the Institute of Subtropical Crops, Nanning, Guangxi Province. After 30 d of growth in Hoagland nutrient solution without Cd contamination, seedlings of uniform size and growth, with a plant height of approximately 15 cm, were selected for use. Exogenous CdCl_2_·2.5H_2_O was added to the sisal seedlings by hydroponics for Cd stress. Six concentration gradients were set: 0, 20, 40, 60, 80, and 100 mg/kg. Three sets of replicates were set up for each group of experimental treatments, with five plant replicates for each group. After 14 d, half of the seedlings were harvested and used to determine morphological indexes and Cd content. The other half was washed with deionized water, promptly placed in liquid nitrogen for quick freezing, and stored at -80°C for determination of physiological indexes and transcriptome analysis.

### 2.2 Determination of Cd content in the leaf and root system of sisal

Determination of Cd in sisal roots and leaves was achieved with an inductively coupled plasma emission spectrometer (Agilent 7500a, USA) [23]. The fresh plant samples were killed at 120°C for 1 h and then placed in an oven at 70°C for continuous drying until the dry weight was constant. The samples were ground and mixed. A certain amount of dry samples was taken, left overnight with nitric acid, and then cooked in a digester at 180°C for 3 h. Hydrogen peroxide was added, and cooking was continued for 1 h until the digesting solution was clear and transparent. After standing and cooling, the solution was poured into a 25 mL volumetric flask (the flask was soaked with 10% HNO_3_ for 12 h in advance and washed with pure water four times); finally, the solution was fixed with deionized water up to the scale, and the concentration of Cd in the sample was determined on the spectrometer [24].

### 2.3 Analysis of antioxidant enzymes

The activities of superoxide dismutase (SOD), catalase (CAT), and peroxidase (POD) were ascertained using kits purchased from the Nanjing Jiancheng Institute of Biological Engineering. The enzymes were prepared as follows. Catalase (CAT): Weigh 0.1g of the fresh sample, add 0.9 mL of physiological saline for grinding, grind until homogenized, centrifuge at 2500 rmp/min for 10 min, and take the supernatant night. Take 0.05mL of the supernatant, add 2.2mL of the reaction mixture, and react for 1 min at 37°C. The extracted CAT enzyme was measured by UV spectrophotometer and its absorbance value was determined at 405 nm. Superoxide Dismutase (SOD): Weigh 0.1 g of the fresh sample, add 0.9 mL of phosphate buffer (0.1 mol/L pH 7) for grinding, grind until homogenized, centrifuge at 3500 rmp/min for 10 min, and take the supernatant night. Take 20 μL of the supernatant, add 220 μL of the reaction mixture, react at 37°C for 20 min, and measure the absorbance value at 450 nm using an enzyme standard. Peroxidase (POD): Weigh 0.1 g of the fresh sample, add 0.9 mL of phosphate buffer (0.1 mol/L pH 7) for grinding, grind until homogenized, centrifuge at 3500 rmp/min for 10 min, and take the supernatant night. Take 0.1 mL of the supernatant, add 3.9 mL of the reaction mixture, react at 37°C for 30 min, and measure the absorbance value at 420 nm with a UV spectrophotometer.

### 2.4 Transcriptome sequencing and data analysis

Total RNA samples were assayed and enriched for eukaryotic mRNA using magnetic beads with Oligo (dT). The obtained mRNA was randomly interrupted and the first strand of cDNA was synthesized in the M-MuLV reverse transcriptase system. The second strand of cDNA was synthesized under the DNA polymerase I system. The cDNA fragments of approximately 370–420 bp in size were screened for cDNA amplification, and the purified products were purified using AMPure XPbeads. The purified products were then subjected to library construction. The insert size of the library was checked with the Agilent 2100 bioanalyzer. To ensure the quality of the library, its effective concentration was checked by the qRT-PCR method. After the libraries were qualified, Illumina NovaSeq 6000 sequencing was performed, and 150 bp paired-end reads were generated.

Before assembly, data quality control analysis was performed for quality control of the raw data (raw data) by filtering the sequencing fragments (reads) from the machine, removing the reads with adapters, the N-containing reads, and the low-quality reads to obtain high-quality data. The clean reads were then spliced using the Trinity (v2.6.6) software. The GOseq (1.10.0) and KOBAS (v2.0.12) software was employed for GO functional enrichment analysis and KEGG pathway enrichment analysis on the differential gene sets.

### 2.5 RNA extraction, reverse transcription, and qRT-PCR

Total RNA was extracted from sisal roots using the TaKaRa MiniBEST Plant RNA Extraction Kit. The extracted total RNA was detected by micro UV-Vis spectrophotometer. Selected RNA samples with absorbance OD_260_/OD_280_ between 1.8 and 2.0 were then used for subsequent qRT-PCR experiments. The extracted RNA was reverse transcribed with the HiFicript gDNA Removal cDNA Synthesis Kit to obtain cDNA which was employed as a template for the qRT-PCR experiments. Quantitative real-time PCR was performed with the SYBR Premix Ex Taq kit (TaKaRa) and the QuantStudio Real-Time PCR System detection system. Eight differential genes (four up-regulated and four down-regulated genes) identified in the re-roots were randomly selected for specific primer design using Primer 5.0. Information on the specific primers utilized in this study as listed in S1 and S2 Tables. The Primer design was completed and sent to UW Genetics for primer synthesis. The look-alike gene for fluorescence quantification was selected as the sisal actin gene that was sequenced and validated by Shenzhen UW Genetics. The Real-time PCR run protocol was as follows: pre-denaturation at 95°C for 30 s, denaturation at 95°C for 30 s, annealing at 60°C for 30 s, extension at 72°C for 30 s, and 40 cycles. Each sample included three biological replicates and three technical replicates. The 2^-ΔΔCT^ method was used for relative expression calculation.

### 2.6 Statistical analysis

All data from this experiment were obtained by repeating the results more than three times, and statistical analysis was performed with the SPSS 25 software. The data obtained from the experiment were tested for significance and one-way analysis of variance was performed using Duncan’s method (p<0.05). Graphs were plotted with Excel 2021 and Prism 5.

## 3. Results

### 3.1 Effect of Cd stress on root growth

**Fig 1:**
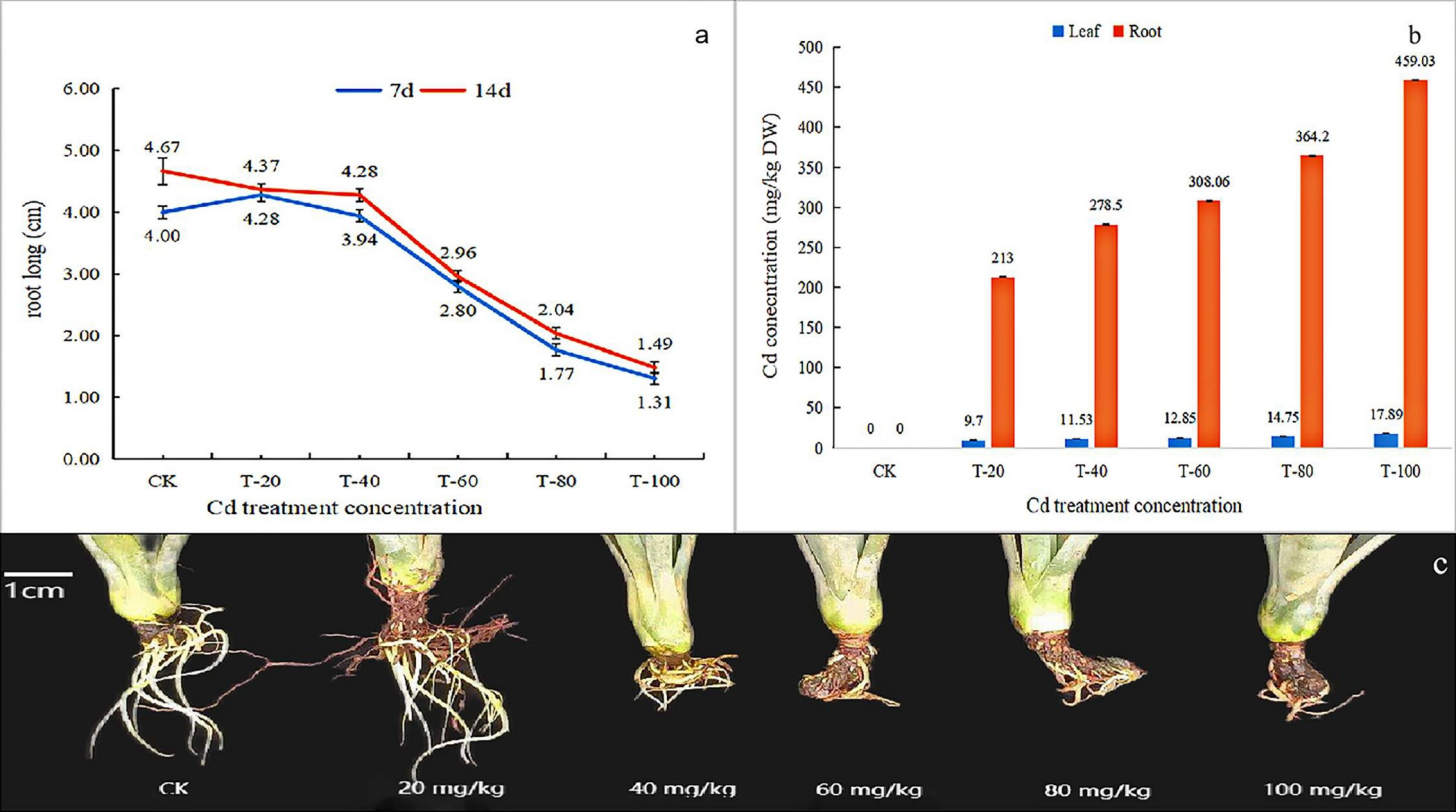
a: Sisal root length at 7 and 14 days of stress; b: Sisal root growth at 14 days of stress; c: Sisal root and leaf Cd content; CK: 0mg/kg; T-20: 20mg/kg; T-40: 40mg/kg; T-60: 60mg/kg; T-80: 80mg/kg; T-100: 100mg/kg.

When Cd stress was applied for 7 d, the average root length of sisal was 4.28 cm at a treatment concentration of 20 mg/kg, which was 0.28 cm longer than that of the control group, indicating that a low concentration of Cd stress for a short time had a promoting effect on sisal root growth (Fig 1A). The root length of sisal was 0.06, 1.2, 2.23, and 2.69 cm shorter than that of the control group at treatment concentrations of 40, 60, 80, and 100 mg/kg, respectively. The root growth of sisal was inhibited by high Cd stress (>20 mg/kg), and the root length of the treated group was shorter than that of the control group. When the treatment duration reached 14 d, Cd stress had a significant inhibitory effect on sisal root growth, and the higher the Cd concentration the stronger the inhibitory effect on the sisal root system. When the treatment concentration was 80 mg/kg, the root length of sisal in the control group was 2.28 times longer than that in the treatment group, and when the treatment concentration was 100 mg/kg, the root length of sisal in the control group was 3.13 times longer than that in the treatment group (Fig 1A). The effects of different concentrations of Cd stress for 14 d on sisal root length are shown in Fig 1B. When the treatment concentration was 60 mg/kg, the sisal seedling root system showed browning. When the concentration reached 80 and 100 mg/kg, the root system of sisal seedlings decayed (Fig 1B), suggesting that the root cells were severely poisoned, resulting in cell death.

### 3.2 Cd accumulation in sisal roots and leaves

The Cd content in sisal roots and leaves increased with the increase of Cd concentration, and the Cd enrichment in roots was significantly higher than that in leaves (Fig 1C). When the Cd stress concentration was 100 mg/kg, the maximum amount of Cd accumulated in sisal roots reached 459.03 mg/kg, and the Cd content in leaves was 17.89 mg/kg. Meanwhile, the concentration and accumulation trend in sisal under each concentration of Cd stress followed the order root > leaf. The accumulated Cd in sisal root was 21.9–25.7 times than that in leaves. Thus, sisal roots are the main organs for Cd enrichment.

### 3.3 Changes in the activity of antioxidant enzymes

**Fig 2:**
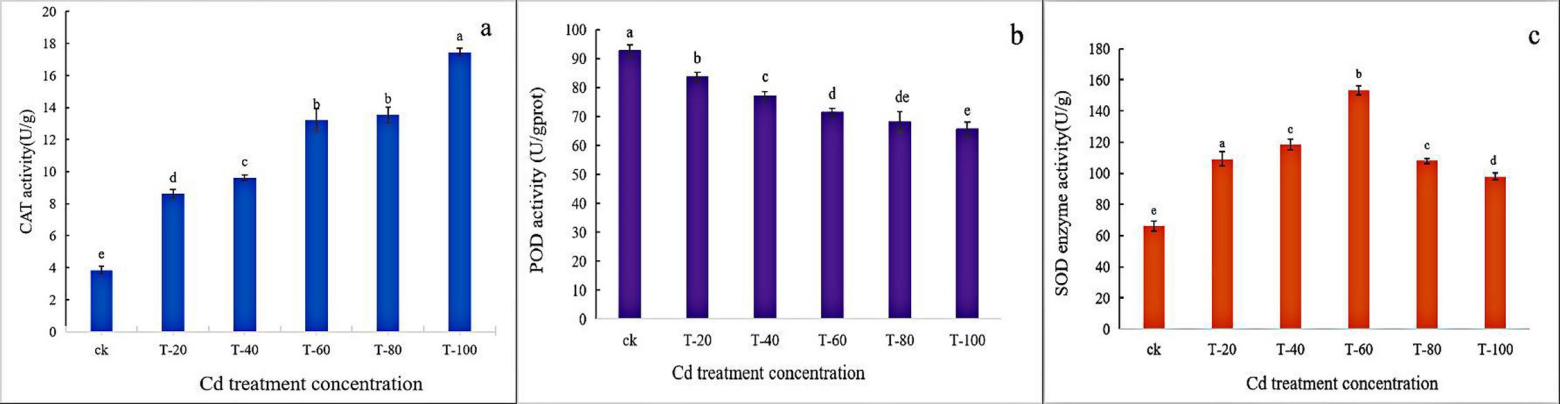
Effect of Cd on CAT (a), POD (b), and SOD (c) activities in sisal roots; Different letters in the same graph indicate significant differences (P<0.050) and the same letters indicate non-significant differences (P>0.050).

CAT plays a key role in scavenging H_2_O_2_ from plants. The changes in CAT activity of sisal subjected to different concentrations of Cd stress are shown in Fig 2A. CAT activity was enhanced by Cd stress compared to the Cd-stressed control. Moreover, CAT content increased with the increase of Cd concentration and reached the maximum concentration (100 mg/kg). CAT activity also reached a maximum value of 17.46 U/g and was elevated by 22.03% compared to the control.

The changes in the POD activity of sisal roots subjected to different concentrations of Cd stress are shown in Fig 2B. The POD activity showed a decreasing trend with increasing Cd concentration. The said activity revealed a minimum value of 65.67 U/g when the Cd stress concentration reached 100 mg/kg, a value which indicates a decrease of 29.14% compared with the control group.

As to SOD activity, the experimental group showed elevated activity (with a trend of first increasing and then decreasing) relative to the counterpart for the control group (Fig 2C). When the Cd concentration was 60 mg/kg, the strongest SOD activity was 153.1967 U/g, which was 2.3 times higher than that of the control group. Thus, sisal formed a relatively strong antioxidant enzyme protection system to reduce its persecution by Cd. However, the SOD activity decreased when the Cd stress concentration reached 80 and 100 mg/kg, and the H_2_O_2_ and O_2_ in sisal roots might have exceeded the SOD removal capacity. Therefore, sisal roots were persecuted by oxidative stress.

### 3.4 Sequencing results

Sisal root rot occurred at 14 d of Cd stress (Fig 1C), and we believe that 80 mg/kg was a threshold value for Cd stress tolerance in sisal, so a concentration of 80 mg/kg was selected for transcriptome sequencing.

The RNA sequencing results showed clean reads of 6.6 G to 6.8 G for each root sample. The Q20 and Q30 values exceeded 97.26% and 92.02%, respectively (Table 1). These findings indicate that the quality of sequencing data is sufficient for further transcriptome analysis.

**Table 1:** RNA sequencing results.

Sample	Raw reads	Raw bases	Clean reads	Clean bases	Errorrate	Q20	Q30	GC pct
**C_1**	23096110	6.9	22724723	6.8	0.03	97.1	92.2	47.82
**C_2**	27903613	8.4	27356500	8.2	0.03	97.03	92.18	48.67
**C_3**	22685308	6.8	22077605	6.6	0.03	97.32	92.76	48.18
**T_1**	22947305	6.9	22142746	6.6	0.03	96.98	92.04	49.23

Sample: Name of sample; raw reads: Number of reads in the original data; raw bases: Number of bases in the original data (raw base = raw reads*150bp); clean reads: Number of reads after raw data filtering; clean bases: Number of bases after raw data filtering (clean base = clean reads*150bp); error rate: Overall sequencing error rate of data; Q20: Percentage of bases with Phred values greater than 20 to total bases; Q30: Percentage of bases with Phred values greater than 30 to total bases; GC pct: Percentage of G and C of the four bases in clean reads

### 3.5 Identification of DEGs

As mentioned, no reference genome sequence is available for sisal. Therefore, we used the Trinity software to de novo assemble all 223735211 clean reads to generate unique homologous sequences. A total of 77835921 unigenes were obtained, with an N50 unigene size of 1778 nt and an average length of 963 nt. Approximately 55.75% of these unigenes could be annotated in the NR, NT, Swiss-Prot, KOG, PFAM, GO, and KEGG databases.

**Fig 3:**
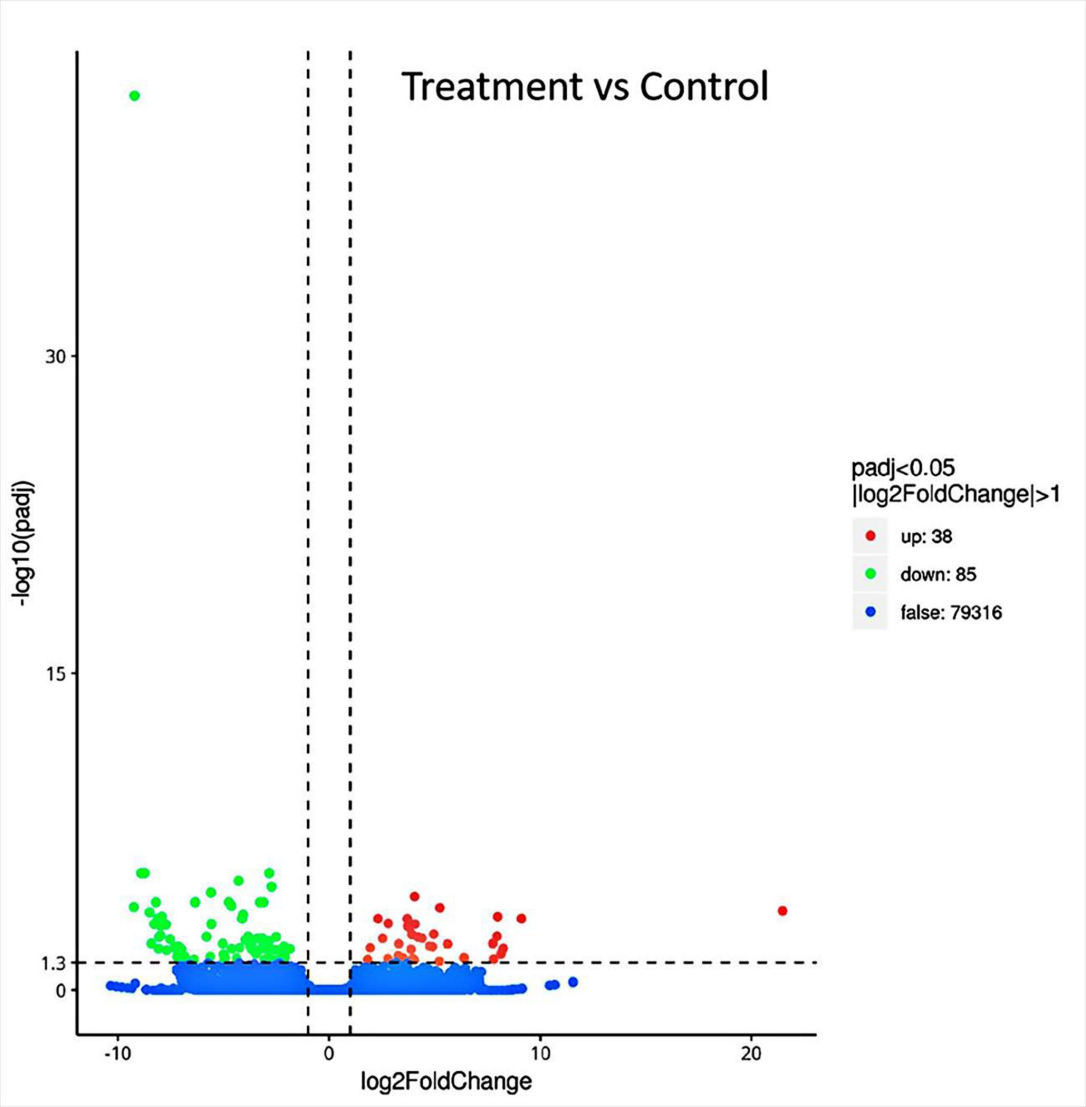
Statistical plot of differential genes. Red indicates up-regulated genes and green indicates down-regulated genes.

For the analysis, the criteria for considering differential gene expression entailed a 2-fold change or greater (|log_2_ fold-change|> 1) and q < 0.05. On the basis of sample expression levels, the samples were tested for differential genes, and the results are shown in Fig 3. A total of 123 DEGs were used in sisal roots at a Cd stress concentration of 80 mg/kg, of which 85 DEGs were down-regulated and 38 DEGs were up-regulated. The differences in gene expression suggest that differential genes play a direct or indirect role in the defense and detoxification of sisal against Cd.

### 3.6 DEGs GO analysis

**Fig 4:**
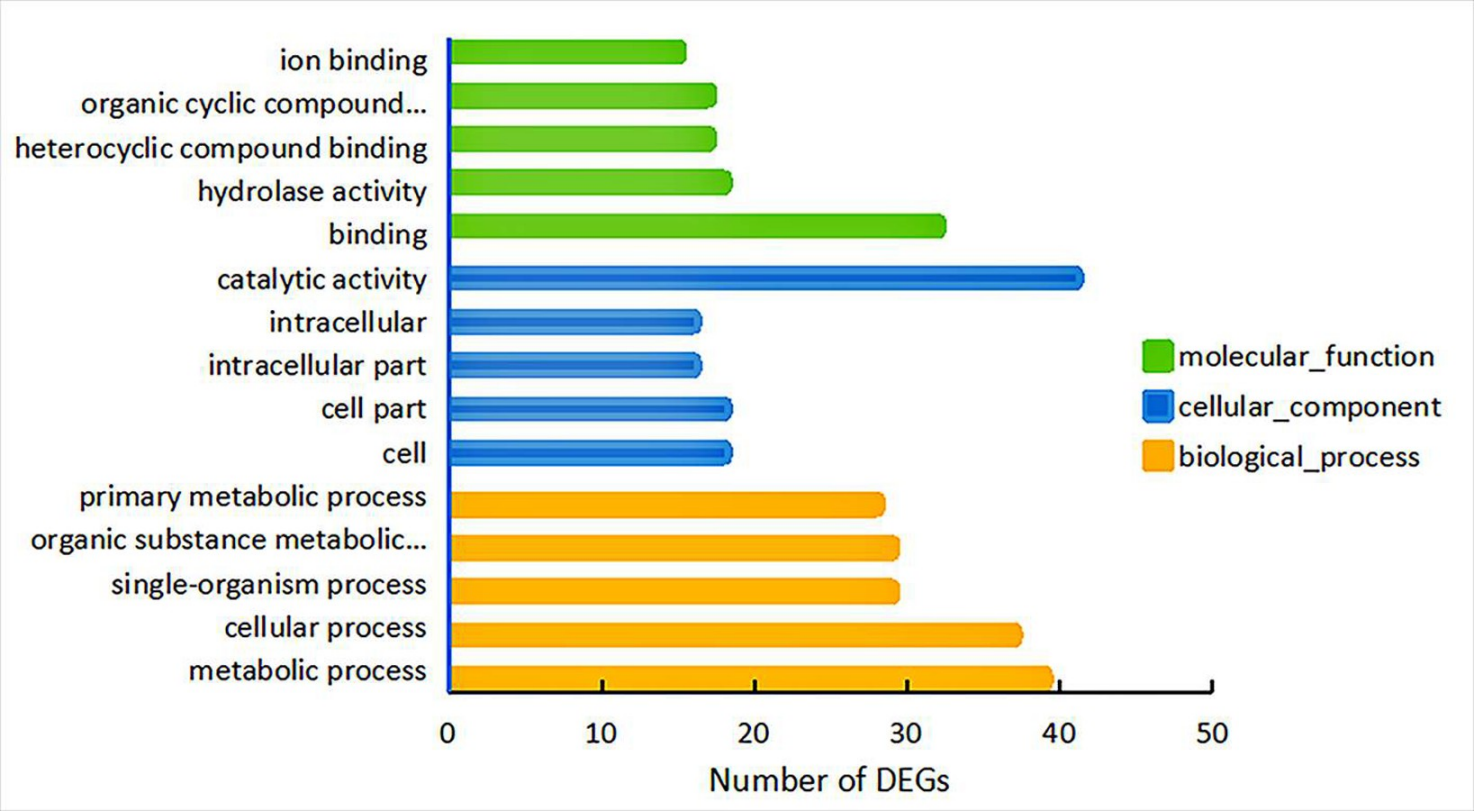
GO functional annotation statistics of differential genes in the sisal root system under Cd stress.

GO was divided into three major functional categories, namely biological process, cellular part, and molecular function. The top five GO functional annotations with the highest percentages are listed in Fig 4. In the differential gene expression of sisal roots, the highest proportion of biological process types was for the metabolic, followed by the cellular process. The highest proportion of the cellular part was for catalytic activity, followed by the cell and intracellular parts. Among the types of molecular functions, the highest proportion involved binding, followed by hydrolase activity.

**Fig 5:**
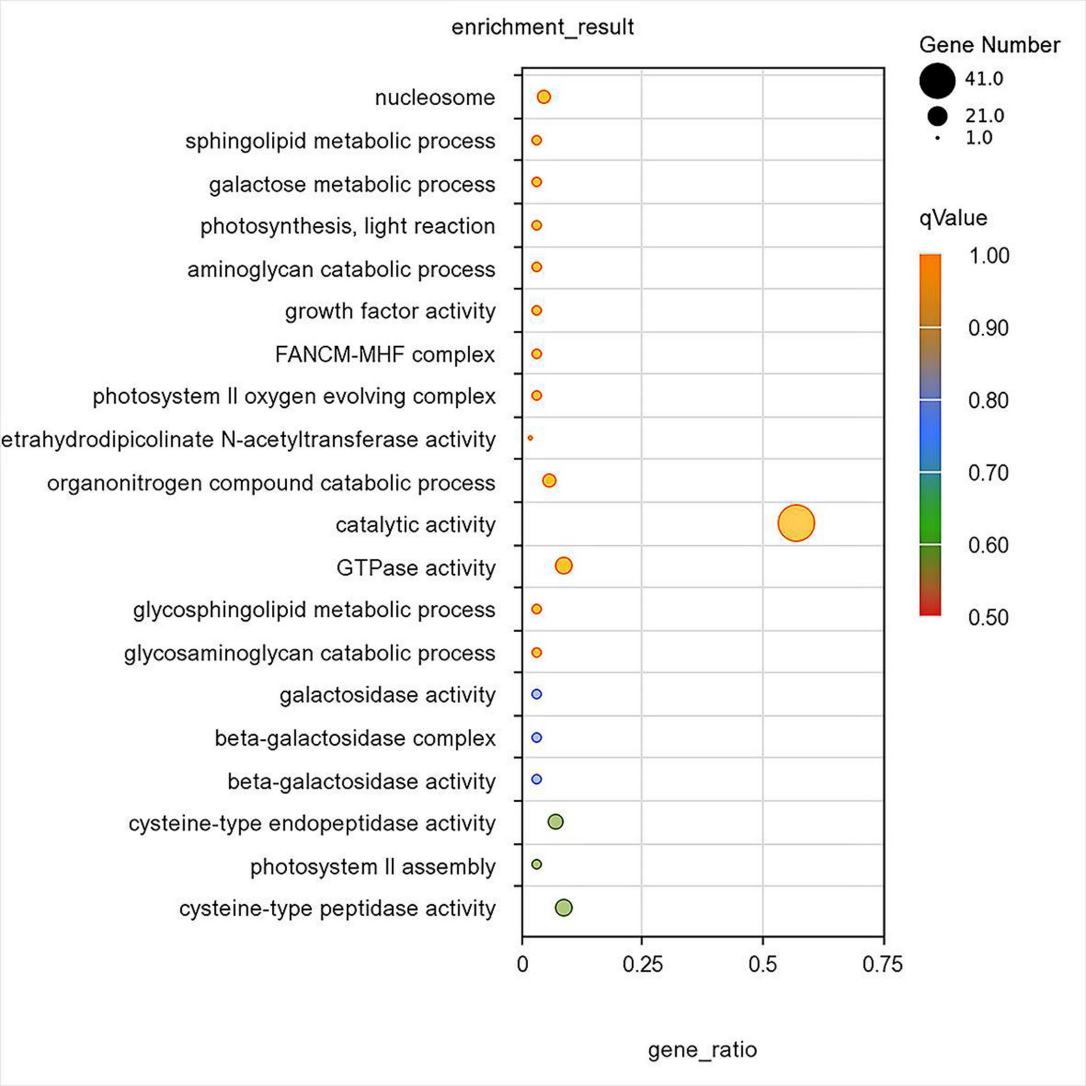
Differential gene GO enrichment analysis.

GO enrichment analysis of differentially expressed genes revealed that the GO function of the sisal root system under 80 mg/L Cd treatment was mainly enriched in the photosystem II assembly (GO:0010207), cysteine-type peptidase activity (GO:0008234), and cysteine-type endopeptidase activity (GO:0004197) (Fig 5).

### 3.7 KEGG metabolic pathway enrichment analysis

**Fig 6:**
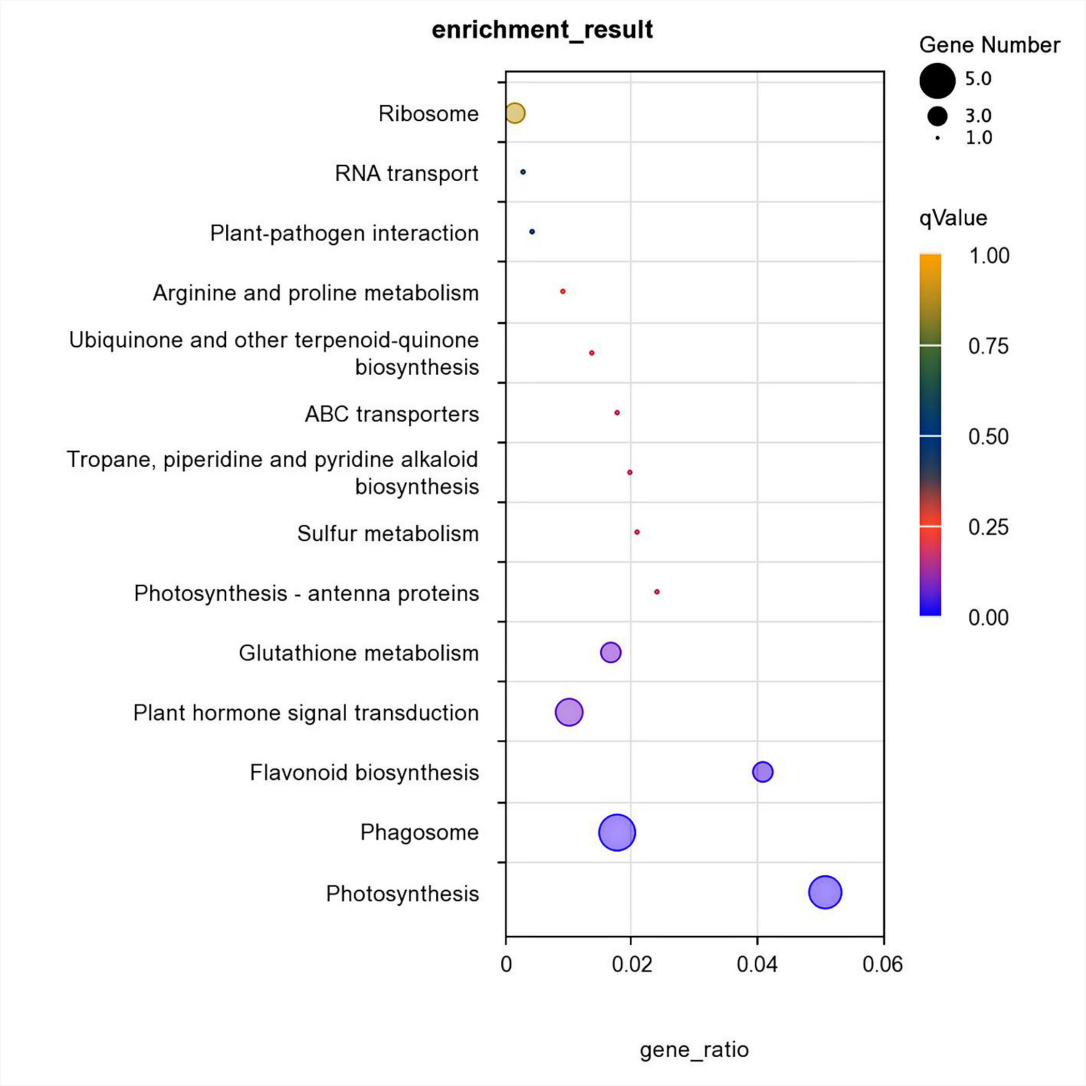
Classification map of the pathways of differential genes.

Different genes in roots coordinate with one another to perform their respective biological functions, and pathway analysis can help to further understand the biological functions of the genes. The KEGG metabolic pathway analysis of differentially expressed genes in sisal roots under Cd stress helped reveal the molecular mechanism of defense and detoxification of Cd in sisal. As shown in Fig 6, the KEGG metabolic pathway enrichment analysis revealed the metabolic effects of Cd stress on sisal through the five metabolic pathways of photosynthesis, phagosome, flavonoid biosynthesis, plant hormone signal transduction, and glutathione (GSH) pathways. The five metabolic pathways were enriched with a proliferation of differential genes. Therefore, secondary metabolism is important for the physiological response of plants when they are exposed to heavy metal stress.

### 3.8 Gene expression validation

**Fig 7:**
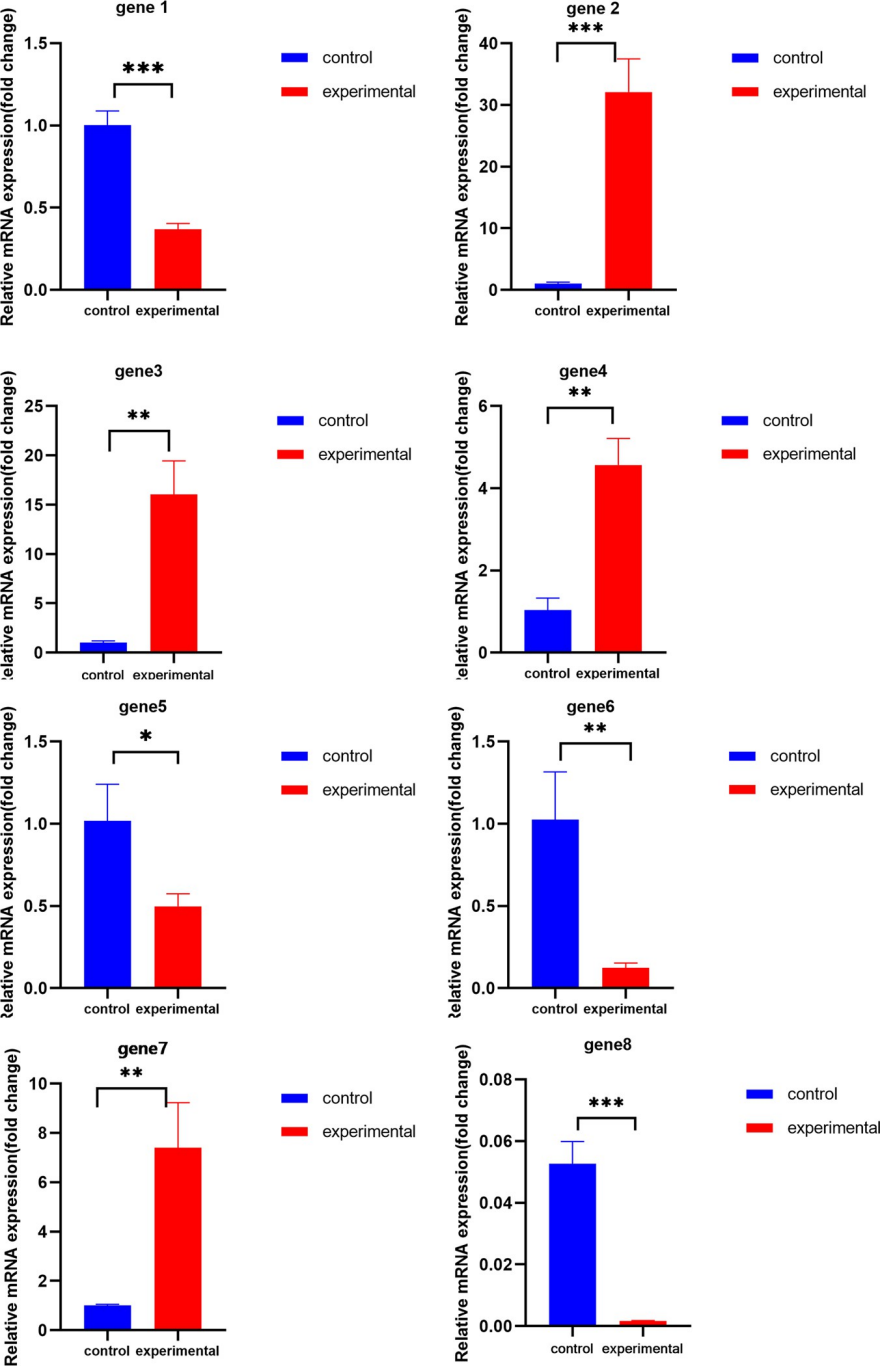
qRT-PCR analysis. Randomly selected eight differential genes and calculated relative gene expressions by qRT-PCR using the 2^-ΔΔCt^ method. Bar graphs indicate the fold difference in transcript levels between the sisal and control under Cd stress; * indicates P<0.05; ** indicates P<0.01; *** indicates P<0.001.

The results of the transcriptome data analysis were validated using qRT-PCR. Eight differentially expressed genes were randomly selected. Details of these eight genes are given in Supplementary Material S1 and S2 Tables. Despite some differences in the extent of the up-or down-regulation of the expression detected by RNA-seq and qRT-PCR, the expression trends of genes reflected by the qRT-PCR results were consistent with the transcriptome sequencing results (Fig 7), an outcome which might be related to the detection range and expression calculation of the two methods. The RNA-seq sequencing results were proven reliable and could represent the differences in transcriptome levels in sisal root systems caused by Cd stress.

## 4. Discussion

The root system has a certain morphological plasticity that enables plants to survive in adverse environments and compete effectively for resources [25]. Cadmium contamination has a considerable effect on root morphogenesis that varies depending on the crop species and the degree of contamination [26]. We investigated the early effects on the sisal root system under high Cd stress. The average root length of sisal was longer than that of the control group at a Cd stress concentration of 20 mg/kg. Moreover, the root growth of sisal was inhibited at a stress concentration greater than 20 mg/kg, and the average root length was generally shorter than that of the control group. Therefore, short-term Cd stress had a “low promotion, high inhibition” effect on sisal root length, similar to the results of previous studies [27]. Concentration analysis showed that Cd mainly gathered in sisal roots, and only a small fraction was transported to the leaves. Therefore, sisal has some resistance to Cd ions and can grow in an environment with a certain amount of Cd stress.

SOD, CAT, and POD are the most important enzymes in the antioxidant enzyme system that control the accumulation of reactive oxygen species in plants. SOD serves to catalyze the binding of the superoxide anion radical (O^-^_2_) to H^+^ to generate H_2_O_2_ and O_2_; POD and CAT further convert H_2_O_2_ to H_2_O, thereby reducing the damage to plant cells from reactive oxygen species [28, 29]. In the present study, SOD and CAT activities increased with the increase of Cd ion concentration, and these occurrences could effectively scavenge ROS and maintain intracellular oxidative balance, consequently mitigating the toxic effects of Cd on plants. Mohamed et al. (2012) confirmed that higher antioxidant enzyme activity in *B. juncea* provided greater detoxification efficiency and gave the plant species better resistance to oxidative stress induced by heavy metals [30]. Moreover, POD activity in this work was decreased with increasing Cd concentration, and a down-regulated gene involved in encoding POD was found in transcriptome analysis, in line with the above biochemical analysis results (Fig 2B).

Transcriptome analysis revealed 123 DEGs in the sisal root system, of which 85 DEGs were down-regulated and 38 DEGs were up-regulated. The variations in gene expressions suggest that differential genes play a direct or indirect role in the defense and detoxification process of sisal against Cd.

In GO functional enrichment, the response to Cd stress strength is first transduced by the biological process of the cellular component response signaling transcriptional response, and then with a sequentially decreasing signal is transduced to membrane components for catalytic oxidation and other biological functions to complete the plant’s Cd stress response to build a resistance phenotype, a finding which is consistent with those of Yang Lanpeng et al. (2017) on the phenotype of *Koelreuteria Paniculata* and the migration of Cd ions [31].

KEGG enrichment results showed that flavonoid biosynthesis and GSH metabolism were the main enrichment metabolic pathways. Flavonoids enhance cellular activity. The uptake of Cd by the roots of sea olive (*Avicennia marina)* was also increased by the addition of flavonoids [32]. Moreover, flavonoids chelate with heavy metals, a reaction which not only catalyze the activity of transport proteins on the cell membrane, but also bind Cd ions to improve Cd tolerance [33]. GSH is a c-Glu-Cys-Gly tripeptide that is important for its triple role as a metal chelator, cellular antioxidant, and ROS signaling molecule in metal detoxification [34, 35]. Further, GSH can act as a metal chelator through its thiol moiety with high metal binding affinity. The expression of GSH synthesis genes GSH1 and GSH2 is induced by Cd treatment in Arabidopsis and contributes to Cd tolerance, and a decrease in GSH levels decreases Cd tolerance [36, 37]. Therefore, flavonoid biosynthesis and GSH metabolism have certain regulatory effects on the Cd tolerance characteristics of sisal, but the specific regulatory mechanisms need further study.

## 5. Conclusions

In this present study, short-term low concentration of Cd stress (20 mg/kg) had a transient promotion effect on the growth of sisal roots, but Cd showed a significant inhibitory effect on the that growth over time. Under different concentrations of Cd stress, the Cd content in sisal was in the order of root > leaf, and Cd accumulated mainly in the root system of sisal.

As for the physiological and biochemical effects of Cd stress on the sisal root system, with the increase of Cd stress concentration, the antioxidant enzyme CAT activity was enhanced,POD activity showed a decreasing trend, and SOD revealed a trend of initial increase and then decrease.

Transcriptome analysis of Cd-stressed sisal identified 123 differential genes, of which there were 85 down-regulated and 38 down-regulated genes. The differential genes were mainly concentrated in flavonoid biosynthesis and glutathione metabolism, and both processes had a regulatory role in the Cd tolerance characteristics of sisal.

However, more comprehensive and clearer mechanisms of Cd response need further investigation. Examples of areas for future research include (1) the isolation and identification of resistance genes, (2) the metabolic regulatory mechanism of Cd tolerance, and (3) the mechanism of Cd^2+^ transport in plants.

## Supporting information

S1 Table Eight differentially expressed genes for qRT-PCR detection. (DOCX)

S2 Table Primers used in the study for quantitative qRT-PCR. (DOCX)
