# Supplementary material for: Exploring cadmium stress responses in sisal roots: Insights from biochemical and transcriptome analysis
Source: PLoS One. 2023 Nov 29;18(11):e0288476. doi: 10.1371/journal.pone.0288476 (PMC10686430; doi:10.1371/journal.pone.0288476)
Supplement: S1 Table — (DOCX) [file pone.0288476.s001.docx]

**S1 Table. Eight differentially expressed genes for qRT-PCR detection**

| **Number** | **Name of gene** |
| --- | --- |
| gene1 | Cluster-24341.26486 |
| gene2 | Cluster-24341.5680 |
| gene3 | Cluster-24341.14945 |
| gene4 | Cluster-24341.36315 |
| gene5 | Cluster-24341.5629 |
| gene6 | Cluster-24341.43777 |
| gene7 | Cluster-25373.0 |
| geng8 | Cluster-27864.1 |
