# Supplementary material for: Exploring cadmium stress responses in sisal roots: Insights from biochemical and transcriptome analysis
Source: PLoS One. 2023 Nov 29;18(11):e0288476. doi: 10.1371/journal.pone.0288476 (PMC10686430; doi:10.1371/journal.pone.0288476)
Supplement: S2 Table — (DOCX) [file pone.0288476.s002.docx]

**S2 Table. Primers used in the study for quantitative RT-PCR**

| **Down regulated gene** | | | |
| --- | --- | --- | --- |
| **Gene ID** | **Name of primer** | **Sequence of primers（5'→3'）** | **Length of product（bp)** |
| Cluster-25373.0 | Forward | 5' CATGACAATTGCTGCTGCTCTTGC3' | 105 |
|  | Reverse | 5'AGGAAACGGTTGACGATTGCTGTAG 3' |  |
| Cluster-27864.1 | Forward | 5' CTGAAGGAGGTGATGGCTAGAATGG 3' | 145 |
|  | Reverse | 5' CGAACAAGGCAACAAGCAAGAGTG 3' |  |
| Cluster-24341.26486 | Forward | 5'GAGTTCTTGGTGCCGAGTTACAGAG 3' | 145 |
|  | Reverse | 5' GGTCCTTGATGTTCTCCTTGCTCAG 3' |  |
| Cluster-24341.43777 | Forward | 5' AGCCACCACCTCCAGGAATACTTC 3' | 133 |
|  | Reverse | 5' AGTAGACCTCGCTCAGGCAATCG 3' |  |
| **Up-regulated gene** | | | |
| **Gene ID** | **Name of primer** | **Sequence of primers（5'→3'）** | **Length of product(bp)** |
| Cluster-24341.5680 | Forward | 5' TCTCTGCGTCCTGGACTCAAGC 3' | 125 |
|  | Reverse | 5'CCTCCTCATCAAGCACTCCTCCTC 3' |  |
| Cluster-24341.5629 | Forward | 5' AAGGTGTCTGTTGGTGTGCTTGG 3' | 150 |
|  | Reverse | 5' GTCGCCGTAGTGAGTGGTTTCG 3' |  |
| Cluster-24341.14945 | Forward | 5' GCAGCGAAGGAGGCAATGAAGG 3' | 112 |
|  | Reverse | 5' TTTGAAGCAGCAGAGGTGTAGTGTC 3' |  |
| Cluster-24341.36315 | Forward | 5' GTCATAGGGCACTTAGGCACACAC 3' | 144 |
|  | Reverse | 5' TCATCCCTTCTTGGTCCACTCTCC 3' |  |
